# Impact of silver substitution on the structural, magnetic, optical, and antibacterial properties of cobalt ferrite

**DOI:** 10.1038/s41598-023-41729-7

**Published:** 2023-09-21

**Authors:** Waqia Tahir, Talat Zeeshan, Salma Waseem, Muhammad Danish Ali, Zohra Kayani, Zill-e-Huma Aftab, Syed Muhammad Talha Mehtab, Safa Ezzine

**Affiliations:** 1 Department of Physics, Lahore College for Women University, Lahore, Pakistan; 2 Institute of Physics Centre for Science and Education, Silesian University of Technology, Krasinskiego 8A, 40-019 Katowice, Poland; 3 Ph.D. School, Silesian University of Technology, 2a Akademicka Str., 44-100 Gliwice, Poland; 4 University of the Punjab, Lahore, 54590 Pakistan; 5 Kyiv Medical University Ukraine, Kyiv, Ukraine; 6 Department of Chemistry, College of Sciences Abha, King Khalid University, Abha, Kingdom of Saudi Arabia

**Keywords:** Engineering, Nanoparticles, Organic-inorganic nanostructures, Structural properties, Materials science, Synthesis and processing

## Abstract

Silver-doped Cobalt Ferrite nanoparticles Ag_x_Co_1−x_Fe_2_O_4_ with concentrations (x = 0, 0.05, 0.1, 0.15) have been prepared using a hydrothermal technique. The XRD pattern confirms the formation of the spinel phase of CoFe_2_O_4_ and the presence of Ag ions in the spinel structure. The spinel phase Ag_x_Co_1−x_Fe_2_O_4_ nanoparticles are confirmed by FTIR analysis by the major bands formed at 874 and 651 cm^−1^, which represent the tetrahedral and octahedral sites. The analysis of optical properties reveals an increase in band gap energy with increasing concentration of the dopant. The energy band gap values depicted for prepared nanoparticles with concentrations x = 0, 0.05, 0.1, 0.15 are 3.58 eV, 3.08 eV, 2.93 eV, and 2.84 eV respectively. Replacement of the Co^2+^ ion with the nonmagnetic Ag^2+^ ion causes a change in saturation magnetization, with Ms values of 48.36, 29.06, 40.69, and 45.85 emu/g being recorded. The CoFe_2_O_4_ and Ag^2+^ CoFe_2_O_4_ nanoparticles were found to be effective against the Acinetobacter Lwoffii and Moraxella species, with a high inhibition zone value of x = 0.15 and 8 × 8 cm against bacteria. It is suggested that, by the above results, the synthesized material is suitable for memory storage devices and antibacterial activity.

## Introduction

In the current era, nanotechnology modifies and plays a vital role in almost every field of human life because of its unique and marvelous electrical, physiochemical, and mechanical effects^1–3^. Nano-sized materials are supposed to be the discrete state of matter, due to their unique and astonishing attributes such as (1) large surface area to volume ratio and (2) quantum effects^4^. These flawless improvements in properties made them suitable for various biomedical applications like targeted drug delivery, MRI (magnetic resonance imaging), cell labeling, gene therapy, cancer treatment, and various medical devices^5–16^. Magnetic nanoparticles have been the focus of interest due to their mesmerizing properties; they may potentially be used in catalysis along with nanomaterials as base catalysts, nano-fluids, and optical filters. The assets of these nanoparticles usually depend upon the fabrication technique and chemical composition^17^. Ferrites are ceramic materials that have a hard and brittle nature^18^. The properties of spinel ferrites are based on various factors, such as the method adopted for material synthesis, time and temperature, the stoichiometric ratio, cationic distributions among tetrahedral and octahedral sites, particle size, and morphology^19^. Nowadays, cobalt ferrite magnetic nanoparticles have a great interest for researchers due to their high coercivity, magneto crystalline anisotropy, chemical stability, moderate saturation magnetization, and morphology^20–22^. To overcome the limitations, raised in using these MNPs such as poor heating efficiency, biocompatibility, etc.; the usability of iron oxide nanoparticles is quite higher because it can be metabolized and transported by proteins easily and successfully used in the pharmaceutical field at the nano-scale. The cubic spinel ferrites (MFe_2_O_4_, where M is a divalent metal ion) are a fundamental type of magnetic materials that have high saturation magnetization and high thermal efficacy^23^. It is well-known that both cobalt and iron are present in the human body, therefore the stability of Co^2+^ in the divalent state and Fe^+3^ in the trivalent state is higher, therefore, the chance of aerial oxidation is less in such materials^24^. CoFe_2_O_4_ is preferably doped with transition metals to intensify the scope of material in biomedical applications such as hyperthermia, magnetic resonance imaging, magnetic separation, drug delivery, biosensors, etc.^25,26^. These nanoparticles are also used as antimicrobic agents against morbific and drug-resistant microbes that constitute the stimulating area of research^27^. Different transition metals such as copper, zinc, nickel, silver, etc. play vital roles in different fields of life. For example, Zinc substituted cobalt ferrite nanoparticles are used to make transducers, transformers, and biosensors as well as antibacterial properties^28^ whereas Nickel doped cobalt ferrite nanoparticles have wide applications in the microwave, high-density recording media, and electronic devices^29^. Silver (Ag) is a transition metal that is both conductive and plasmonic, and its electrical structure permits the development of an electron cloud. These oscillating and light-interacting delocalized electrons can produce unique optical and electrical features^30^. It is the preferred metallic element among those used for electronics, photonics, biological sensing, solar cell surface coatings, catalysts, and staining pigments^31^. Silver (Ag) nanoparticles were chosen as the most favorable metal among all due to their chemical stability, affordability, and highest thermal and electrical conductivity^32^. In the past, antibiotic treatment was considered to be the only way for various bactericidal purposes for saving countless lives. However, several studies have evidence that excessive antibiotic use can cause multidrug-resistant bacterial strains^33^. Numerous factors became the cause of ‘super-bacteria’, such as the use of antibiotics in excess quantity, low quality, and wrong prescriptions. To overcome this fatal situation for global healthcare, various nanoparticles have been studied for antibacterial activity^34,35^. In ancient civilizations, silver and its colloidal suspensions are usually used to diminish infectious disorders. Feasible antimicrobial mechanisms have been involved in microbial killing actions by Ag nanoparticles, such as DNA damage, disruption of the bacteria cell membrane, release of silver ions, and electron transport^36–38^. These nanoparticles with low toxicity and superior oligodynamic performance are preferably used as antimicrobial agents in commercialized consumer goods including diabetic wound dressings, bactericidal coatings on surgical instruments, germicidal soaps, skin lotion, and creams. Ag_x_Co_1−x_Fe_2_O_4_ nanoparticles at the nanoscale are more beneficial for antibacterial activity and the magnetic property of cobalt ferrite nanoparticles helps the material stabilize its magnetic dispersion and makes them more effective and less toxic for human health^39–41^. Due to affordability and extensive compositional control, the hydrothermal method is one of the most widely utilized techniques.The nucleation and morphological growth rate of the crystals during the hydrothermal process regulates the size of the crystallizing particles^42^. Palak Mahajan et al.^43^ studied Ag_x_Co_1−x_Fe_2_O_4_ nanoparticles' antibacterial activity and concluded that it is more effective against gram-positive bacterial strains in comparison with gram-negative bacterial strains. Okasha et al.^44^ analyzed the variations caused by Ag doping in MgFe_2_O_4_ and described its thermal and electrical conductivity. M.K. Satheeshkumar et al.^45^ examined the magnetic, structural, and bactericidal properties of Ag_x_Co_1−x_Fe_2_O_4_ nanoparticles, revealing good results of antibacterial activity against some bacteria’s i.e., *Staphylococcus aureus*, *Escherichia coli*, and *Candida ablicans*.

The reason behind the present work to be adopted is the stability, less toxicity, and efficacy of Ag_x_Co_1−x_Fe_2_O_4_ nanoparticles amongst various bacteria that have been prepared by hydrothermal technique with various concentrations of Ag^2+^ (x = 0, 0.05, 0.1, 0.15) to study its structural, optical, magnetic and bactericidal efficacy against gram-negative bacteria.

## Material and methods

### Chemical and reagents

Cobalt (II) nitrate hexahydrate (Co(NO_3_)_2_.6H_2_O)Riedel-deHaёn, Iron (III) nitrate nonahydrate (Fe(NO_3_)_3_.9H_2_O) UNI-CHEM, Silver nitrate hydrate (AgNO_3_.H_2_O)Sigma-Aldrich, Sodium Hydroxide (NaOH) Riedel-deHaёn, Absolute Ethanol (C_2_H_5_OH) Sigma-Aldrich, and distilled water were used for the synthesis of Ag_x_Co_1−x_Fe_2_O_4_ nanoparticles.

### Synthesis of Ag_x_Co_1−x_Fe_2_O_4_ nanoparticles

**Figure 1:**
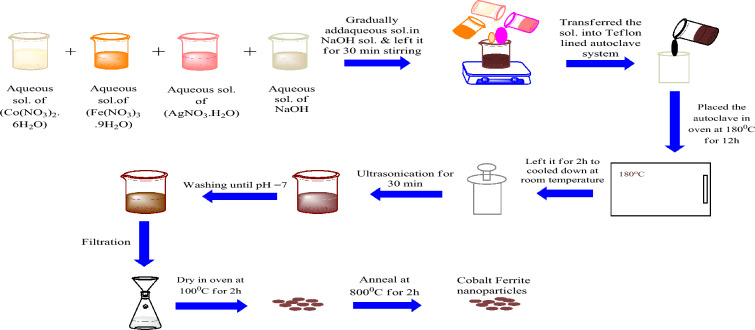
Schematic representation of the Co_1−x_Ag_x_Fe_2_O_4_ nanoparticle synthesis procedure.

Silver-doped cobalt ferrite nanoparticles (Ag_x_Co_1−x_Fe_2_O_4_) with a series of concentrations (x = 0, 0.05, 0.1, 0.15) are prepared by the hydrothermal method. Firstly, 0.5 mol of each nitrate are weighed. The aqueous solution of each nitrate was then prepared with 30 mL distilled water and left for magnetic stirring until the fine solutions of each nitrate were obtained. Next, to maintain the pH of the solution, the aqueous solution of 2 mol of sodium hydroxide is gradually added to the combined solution of cobalt nitrate and iron nitrate under moderate stirring for 30 min at room temperature. Subsequently, this solution was transferred to a Teflon-lined autoclave system and this autoclave system was placed into an oven at 180 °C for 12 h. Finally, the mixture was cooled to room temperature inside the furnace. For homogenized distribution of particles, the obtained precipitates were put in an ultra-sonication bath for 30 min. After ultrasonication, the nanoparticles were washed several times with deionized water and ethanol until the pH was maintained at 7 and the filtrate. Furthermore, these nanoparticles were then placed in an oven for drying at 100 °C for 2 h. Finally, the obtained nanoparticles were put into the furnace for calcination at 800 °C for 2 h. A similar procedure was used for the synthesis of silver-doped cobalt ferrite nanoparticles by adding one more salt of silver nitrate with the mentioned concentrations. The complete process is explained in Fig. 1.

### Preparation for antibacterial activity

To avoid contamination, precautionary measures have been taken: firstly, the hands and Laminar Flow Chamber were sterilized by employing ethyl alcohol. For about 20 min at 121 °C, the Petri plates were also sterilized via autoclaving. A medium was developed to isolate the microflora from LBA (Luria Bertani Agar).

Luria Bertani Agar media (LBA) has been prepared by utilizing the following reagents: (a) 2.5 g yeast extract, (b) 2.5 g NaCl, (c) 5 g tryptone, and (d) 7.5 g agar; and all of these components were then dissolved in 500 ml distilled water and the medium was autoclaved at 121 °C for about 15–20 min and 15psi for sterilization purpose. After that, the prepared media was poured into Petri plates and set aside to become solidified. The LBA media has been prepared for the purpose of isolation and refinement of bacterial strains^46^.

### Characterizations

The synthesized samples have been characterized through X-ray diffractometer (X'Pert PRO MPD) using Cu-Kα radiation (λ = 1.54 Å), Fourier transforms infrared spectroscopy (IRTracer-100 FTIR Spectrometer), Ultraviolet–visible spectroscopy (UV = 2800 model, Hitachi Japan, wavelength = 300–900 nm, quartz), Vibrating sample magnetometer (VSM of Lakeshore 7400) and the antibacterial activity test has been taken by well diffusion method.

### Ethics statement

The author =confirms that the article was not published in any journal.

## Results and discussion

### X-ray diffraction analysis

**Figure 2:**
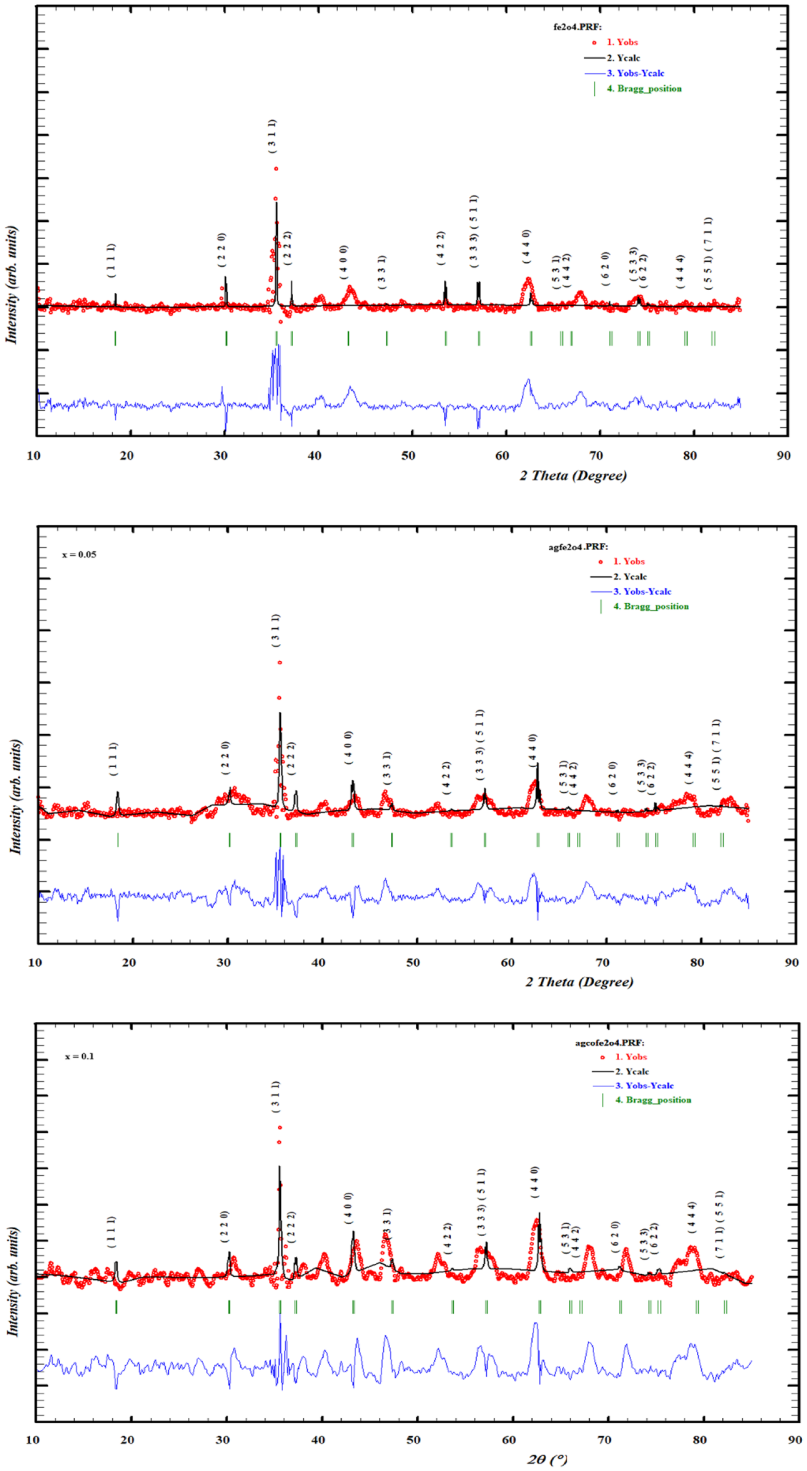

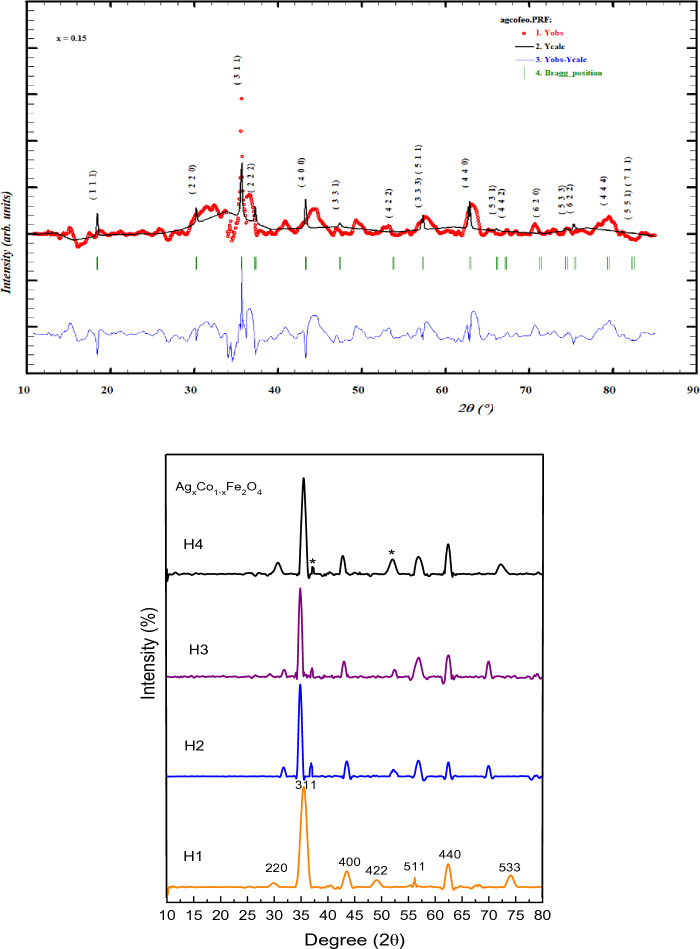
(**a**) Refined graphs with Full Prof software. (**b**) Normalized XRD analysis of different concentrations of Ag with Cobalt ferrites.

**Figure 3:**
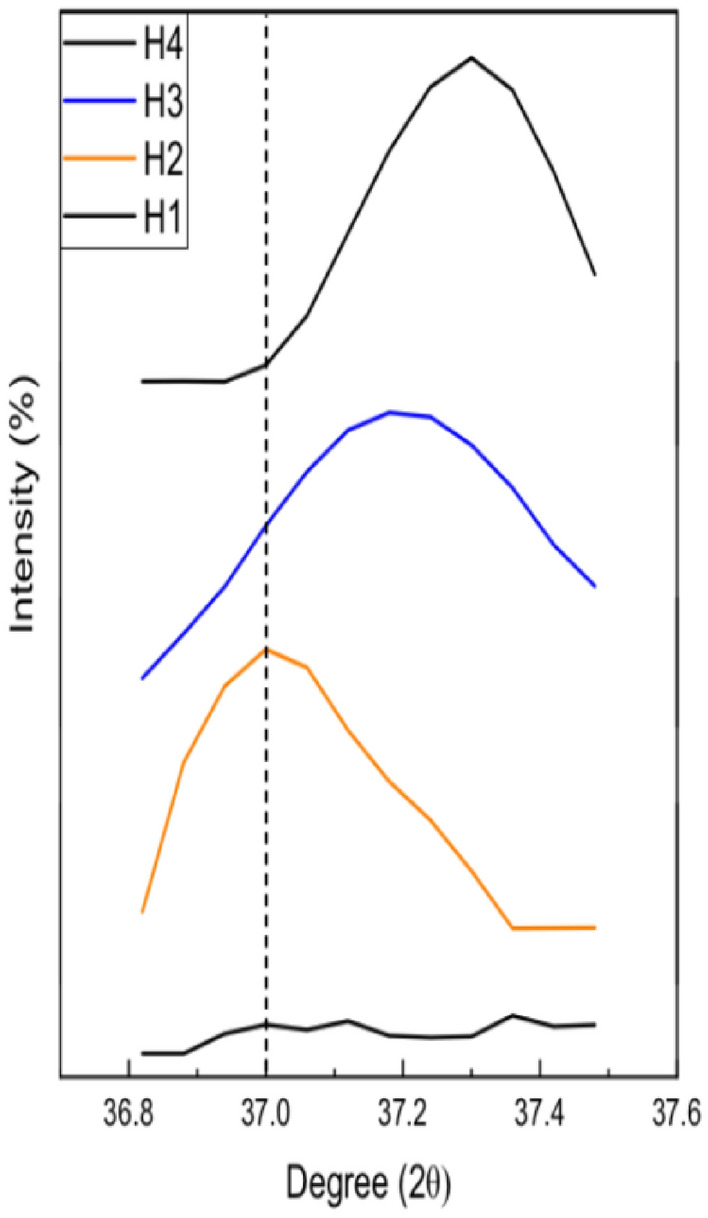
The angle variation against the Ag concentration.

The XRD of silver-doped cobalt ferrite was done with the help of an X-ray diffractometer equipped with Cu-Kα radiation (1.54 Å). The XRD of prepared samples is demonstrated in Fig. 2. The XRD pattern of Ag_x_Co_1−x_Fe_2_O_4_ nanoparticles with various doping ratios like (x = 0, 0.05, 0.1, 0.15) synthesized by hydrothermal technique is figured out in Fig. 2. The overall detected peaks matched accurately with JCPDS card no. 22-1086 with Fd-3 m space group which validates the pattern of cubic spinel structure^47^. Various crystal planes like (220), (311), (400), (422), (511), (440), and (533) are detected with respect to the position of 2θ (30.68, 35.54, 43.42, 53.66, 57.16, 62.39, 74.34)°, respectively. Additional peaks in crystal planes (111), (200) at (37.00, 54.11)° are detected due to the presence of silver at x = 0.05 to onward; this corroborates the presence of metallic silver with JCPDS card no. 04-0783^48,49^. The peak broadening allocates that the particle size of Ag_x_Co_1−x_Fe_2_O_4_ nanoparticles with increasing dopant concentration reaches to nano-regime^50^. The crystalline size of the ferrites has been observed from the literature to be in the range of 04–69 nm^51^. From Fig. 3 it has been observed that there is a minor change in the major silver peak at 37 °C and due to the increment of Ag concentration the height of the peak becomes weak, and it was transferred from 37 to 37.4 °C.

The interplanar spacing of the most prominent peak (311) is calculated by using Bragg’s relation^50,52,53^:

1 $$ {\text{2dsin}}\uptheta \, = {\text{ n}}\uplambda $$

where λ represents the wavelength of Cu–Kα radiation (λ = 1.54 Å) and θ shows the Bragg angle^54^.

The lattice parameter of the samples has been estimated with the help of miller indices (hkl) of most prime peak (311) and the diffraction angle through the following equation^55^:

2 $$\mathrm{a }=\frac{\uplambda \surd ({\mathrm{h}}^{2}+{\mathrm{k}}^{2}+{\mathrm{l}}^{2})}{4\mathrm{sin\,\,\theta }}$$

**Figure 4:**
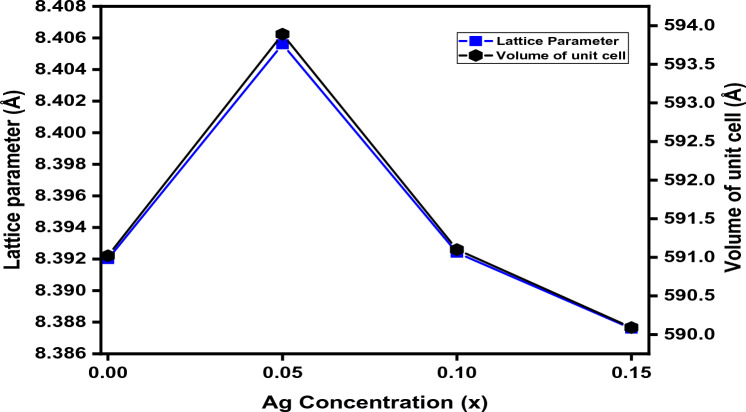
Lattice constant as a function of Ag concentration in Ag_x_Co_1−x_Fe_2_O_4_ nanoparticles.

Figure 4 reveals that the lattice parameter initially increases by the substitution of silver, but after saturation, a decrement behavior is observed in lattice parameters by the increment of silver concentrations. For lower concentrations, it could be expected that the cobalt ions present at the octahedral site are replaced by silver ions and the lattice parameter ‘a’ increases due to the larger ionic radius of silver than cobalt. But on increasing the concentration of silver, the aggregation of Ag^2+^ ions formed at grain boundaries that hindered the further expansion of spinel lattice which caused a decrease in lattice parameter. Moreover, a sudden decrease in lattice parameters could be the compression of the spinel lattice formed at the grain boundaries by the production of a secondary phase due to an excess amount of Ag^2+^^56^.

The Debye’s Scherrer formula is employed to measure the crystalline size^57,58^:

3 $${\mathrm{D}}_{\text{hkl}}=\frac{0.94\lambda }{\beta cos\theta }$$

where λ stands for the wavelength of Cu Kα radiation, 0.94 is a constant value of the shape factor ‘K’, β stands for full-width half maximum ‘FWHM’ of the most extreme peak (311), whereas θ belongs to the diffraction angle^59^. It is observed from Table 1, that the crystallite size of samples varies from 46 to 35 nm, which is confirmation that by increasing the silver content, the crystallite size of nanoparticles is drastically affected. This increase in crystallite size was imputed to peak broadening, which occurs as a result of the strain produced in the unit cell; as a result of the substitution of smaller ionic radii cobalt content with larger ionic radii silver. Furthermore, a rapid decrease in crystallite size occurs because aggregates formed at grain boundaries produce stress in the material; therefore, the crystallite size decreases for higher concentrations of dopant^60^.

**Table 1:** Different parameters related to XRD analysis.

Dopant conc. (x)	0	0.05	0.1	0.15
d-spacing (Å)	2.5303	2.5343	2.5304	2.5289
Lattice parameter(Å)	8.3920	8.4056	8.3924	8.3876
Volume (a^3^)	591.02	593.89	591.10	590.09
Crystallite size (nm)	46.4314	50.5167	35.5058	35.0476
X-ray density (g/cm^3^)	5.2738	5.3031	5.3831	5.4473
Dislocation density	0.00046	0.00039	0.00079	0.00081
Packing factor	18.3501	19.9324	14.0316	13.8584

The X-ray density of the prepared materials has been calculated using the relation given below:

4 $$\mathrm{d }_{\text{x}}= \frac{8M}{N{a}^{3}}$$

where ‘M’ shows the molecular weight of the specimen, ‘N’ represents the Avogadro number and its value is ‘6.023 × 10^23^’, the lattice parameter is denoted by ‘a’, and ‘8’ represents the number of atoms present in a unit cell of cubic spinel structure^61^.

**Figure 5:**
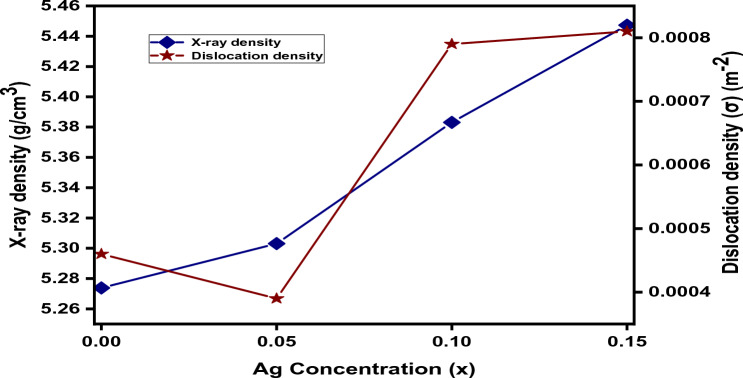
Variation of X-ray density and dislocation density with concentration of Ag in Ag_x_Co_1−x_Fe_2_O_4_ nanoparticles.

It is observed from Fig. 5, that the value of X-ray density shows an increment behavior by increasing the concentration of silver; this is due to increasing the molecular weight by adding the silver which is attributed to the leading increment in molecular weight comparable to that of lattice parameters.

The dislocation density factor has been represented using the following relation:

5 $$\upsigma =\frac{1}{{D}^{2}}$$

where ‘D’ is used for crystallite size, which is determined by Scherrer’s equation^62^.

In Fig. 5, it is concluded that the dislocation density initially decreases and then increases for higher concentrations of Ag^2+^ content. The reason behind the slight increase in dislocation density is the substitution of larger ionic radii Ag^2+^ (1.08Å) with smaller ionic radii Co^2+^ (0.74Å) at the octahedral B-site and the strain produced in the material. The dislocation density decreases with further doping, as the Ag^2+^ ions form aggregates at grain boundaries and the further expansion or dislocation in the spinel lattice is restricted^63,64^.

The relation adopted for the estimation of the Packing factor (P) is given below:

6 $$\mathrm{P }=\frac{D}{d}$$

Here, ‘D’ is used for the crystallite size, and ‘d’ is used for the interplanar spacing^65^.

The packing factor of Ag_x_Co_1−x_Fe_2_O_4_ nanoparticles listed in Table 1 showed that the values slightly increase at an initial stage, but for higher concentrations a sudden decrease is observed; this illustrates that there is no longer dislocation produced in structure by the dopant and the aggregates of the silver ion formed at grain boundaries, due to this these nanoparticles restrict further expansion in the spinel structure. Parameters extracted with the help of an X-ray diffraction micrograph such as; crystallite size, packing factor, FWHM (β), X-ray density, dislocation density, lattice parameter, and d-spacing are listed in Table 1.

Previously using some other techniques, the Ag-CoFe2O4 was produced and various parameters are presented in Table 2:

**Table 2:** The below-mentioned materials were synthesized through the sol–gel method and have various parameters.

Material name	Angle (degree)	Lattice constant (Å)	D (nm)	Density g cm^−3^	Method
Ag_x_Co_1−x_Fe_2_O_4_^100^	35.2	8.3626	18.8		Green method
Ag_x_Co_1−x_Fe_2_O_4_^101^	35.55	8.3721	24.1	–	Combustion method
Ag_x_Co_1−x_Fe_2_O_4_^43^	35.42	8.37	21.6	–	Green synthesis
Ag_x_Co_1−x_Fe_2_O_4_^102^	35.3	8.365	15.01	5.322	Sol–gel
Ag_x_Co_1−x_Fe_2_O_4_^103^	35.2	8.3	10	–	Photo assisted fenton
Ag_x_Co_1−x_Fe_2_O_4_^104^	35.7	–	11	–	Sol–gel

### Theoretical calculation for the cationic distribution

The distribution of cations for the inverse spinel structure is represented by the following relation^66^:

7 $$ \left[ {{\text{Me}}^{{{2} + }}_{{\text{x}}} {\text{Fe}}^{{{3} + }}_{{{1} - {\text{x}}}} } \right]_{{\text{A}}} \left[ {{\text{Me}}^{{{2} + }}_{{{1} - {\text{x}}}} {\text{Fe}}^{{{3} + }}_{{{1} + {\text{x}}}} } \right]_{{\text{B}}} {\text{O}}^{{ - {2}}}_{{4}} $$

With the doping of silver in cobalt ferrite, cationic disorder is observed, which affects various structural parameters like bond length, ionic radii, etc. The results revealed that with the doping of Ag^2+^ in CoFe_2_O_4_ the bond angles are not triggered, but the bond lengths are slightly affected due to the small expansions and contractions that occur at tetrahedral and octahedral sites^67^. The estimated cationic distribution of Ag_x_Co_1−x_Fe_2_O_4_ nanoparticles is illustrated in Table 3.

**Table 3:** Estimated cationic distribution of Ag_x_Co_1−x_Fe_2_O_4_ nanoparticles.

Composition x	Tetrahedral site	Octahedral site
0	Fe_1_	Co_1_Fe_1_
0.05	Co_0.02_ Fe_0.98_	Ag_0.05_ Co_0.93_ Fe_1.02_
0.1	Co_0.08_ Fe_0.92_	Ag_0.1_ Co_0.82_ Fe_1.08_
0.15	Co_0.14_ Fe_0.86_	Ag_0.15_ Co_0.71_ Fe_1.14_

To calculate the radii of the tetrahedral and octahedral sites, the following formulas are used^68^:

8 $$ {\text{r}}_{{\text{A}}} = \left[ {{\text{C}}_{{{\text{Co}}}} \cdot {\text{ r }}\left( {{\text{Co}}^{{ + {2}}} } \right) \, + {\text{ C}}_{{{\text{Fe}}}} \cdot {\text{ r }}\left( {{\text{Fe}}^{{{3} + }} } \right)} \right] $$

9 $$ {\text{r}}_{{\text{B}}} = \frac{1}{2}\left[ {{\text{C}}_{{{\text{Co}}}} \cdot {\text{ r}}\left( {{\text{Co}}^{{ + {2}}} } \right) + {\text{C}}_{{{\text{Ag}}}} \cdot {\text{r}}\left( {{\text{Ag}}^{{ + {2}}} } \right) \, + {\text{ C}}_{{{\text{Fe}}}} \cdot {\text{ r }}\left( {{\text{Fe}}^{{{3} + }} } \right)} \right] $$

**Figure 6:**
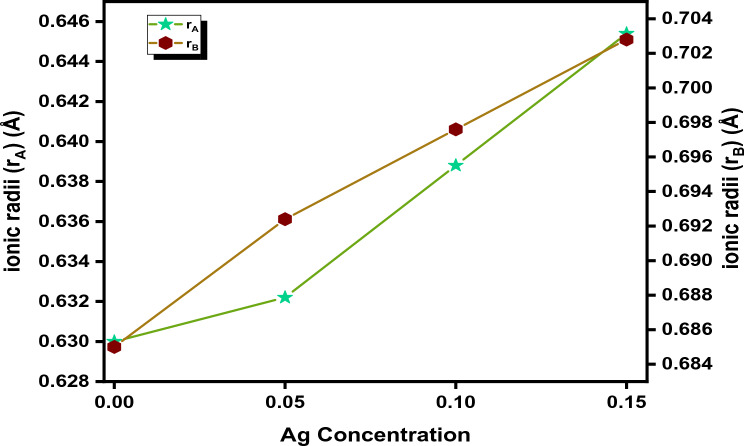
Relation between the ionic radii r_A_ and r_B_ with different Ag concentrations.

According to the proposed cationic distribution; the majority of the ions desired to be doped in place of the Co^2+^ ion present at the B site. Figure 6, illustrates that the values of both r_A_ and r_B_ increase with increasing silver concentration; this might be due to the difference in ionic radii between the substituent and host atoms that leads to an enhancement of the inter-atomic distance which in turn increases the values of r_A_ and r_B._

The lattice constant (a_Th_) is measured theoretically by using the following relation^69^:

10 $$ {\text{a}}_{{{\text{Th}}}} = \frac{8}{3\sqrt 3 }\left[ {\left( {{\text{r}}_{{\text{A}}} + {\text{R}}_{{\text{O}}} } \right) \, + \, \left( {{\text{r}}_{{\text{B}}} + {\text{R}}_{{\text{O}}} } \right)} \right] $$

**Figure 7:**
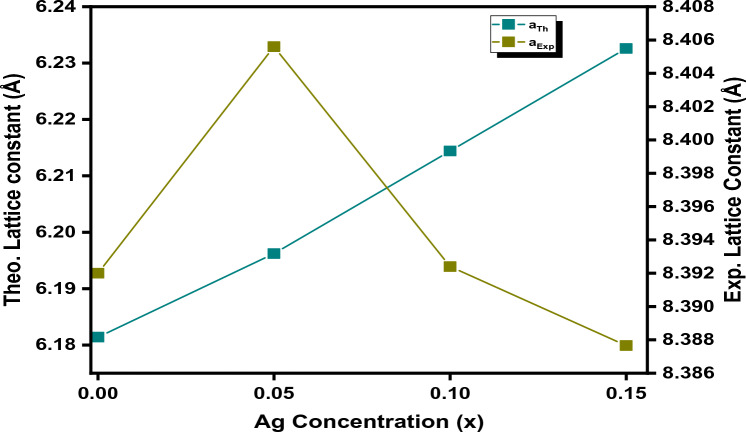
Relation between the theoretical and experimental lattice constants with different Ag concentrations.

Here, ‘a_Th_’ shows the theoretical value of the lattice constant whereas ‘R_0_’ indicates the ionic radii of the oxygen ion. In Fig. 7, we noticed that the experimentally calculated values of the lattice constant are slightly higher than theoretically calculated values. Such deviations in respective values may be due to the formation of secondary phases that cause disorder in the oxygen ions arrangement and subsequently, enlargement or contraction of the tetrahedral and octahedral sites occur due to ion replacement with either trivalent or divalent ions.

Metal atoms of various sizes exhibit voids between oxygen atoms, which becomes the cause of disturbance in close-packed structures. That is why O^2−^ atoms dislocate from their mean position towards the diagonal position. This shift in the O^2−^ atom is termed as ‘u’ (oxygen parameter). Ideally, the value of the oxygen parameter is 0.25, since the ions are arranged in an ideal manner; but in reality, the oxygen parameter has lower values compared to the ideal value^70^. The oxygen parameter ‘u’ can be estimated with the help of the following relation:

11 $$ {\text{u }} = \frac{5}{8} - \frac{{r_{B} + R_{o} }}{a} $$

The values of the parameter u for all prepared materials are illustrated in Table 3.

The deviation observed from the ideal value confirms the substitution of the cobalt ion with silver, which has larger ionic radii in comparison with the preoccupied ion (Co^2+^). The value of the u parameter is observed to slightly increase with the substitution of the Ag^2+^ ion. This fluctuation of the u-parameter from the ideal oxygen parameter occurs due to expansion at the octahedral site. This deviation could be measured by the following relation^71^:

12 $$ \Delta \, = {\text{ u }}{-}{\text{ u}}_{{{\text{ideal}}}} $$

where δ termed an inversion parameter whose calculated values are listed in Table 4.

**Table 4:** Different structural parameters were calculated in order to find the effect of doping on the cationic distribution.

Ag (x)	0	0.05	0.1	0.15
R_Ag_ (Å)	1.08	1.08	1.08	1.08
R_Fe_ (Å)	0.63	0.63	0.63	0.63
R_Co_ (Å)	0.74	0.74	0.74	0.74
r_A_ (Å)	0.63	0.6525	0.675	0.6975
r_B_ (Å)	0.685	0.68225	0.6795	0.67675
R_o_ (Å)	1.35	1.35	1.35	1.35
Tolerance factor	1.0438	1.0455	1.0474	1.0494
Oxygen position parameter (Å)	0.3808	0.3809	0.3811	0.3814
Inversion parameter (δ)	0.0457	0.0478	0.0498	0.0518
a_Th_ (Å)	8.357	8.365	8.389	8.398

To calculate the tolerance factor for Ag_x_Co_1−x_Fe_2_O_4_ nanoparticles, the following equation is used^72^:

13 $$ {\text{T}} = \frac{1}{\sqrt 3 }\left( {\frac{{{\text{rA}} + {\text{RO}}}}{{{\text{rB}} + {\text{RO}}}}} \right) + \frac{1}{\sqrt 2 }\left( {\frac{{{\text{RO}}}}{{{\text{rA}} + {\text{RO}}}}} \right) $$

**Figure 8:**
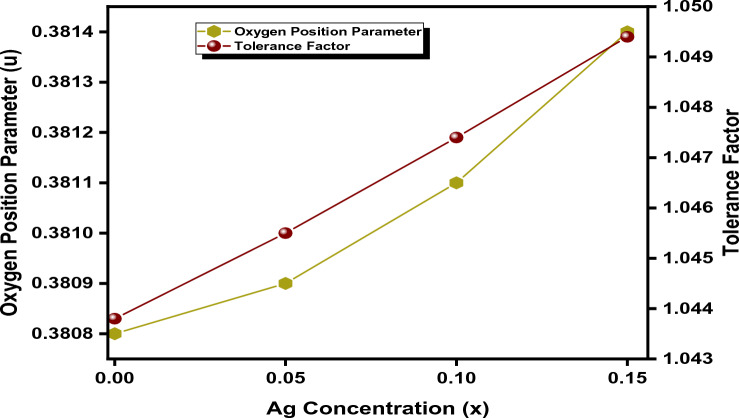
Variation of the oxygen position parameter and the tolerance factor with Ag concentration.

The value of the tolerance factor for all Ag concentrations is tabulated in Table 4. The tolerance factor is near 1 for all concentrations of Ag, indicating that the synthesized sample has a cubic inverse spinel structure. The variation in the u-parameter and tolerance factor with different concentrations of Ag is depicted in Fig. 8.

By measuring the hope length, the interionic distance and spin interaction of magnetic ions could be investigated. The hope lengths ‘L_a_’ and ‘L_b_’ at the tetrahedral and octahedral sites have been calculated by adopting the following relations^73,74^.

14 $$ {\text{L}}_{{\text{a}}} = 0.25{\text{a}}\sqrt 3 $$

15 $$ {\text{L}}_{{\text{b}}} = 0.25{\text{a}}\sqrt 2 $$

The A–A, A–B, B–B cations site and A–O, B–O anions site magnetic interaction depend on their bond lengths and angles. The magnetic strength has a direct relation with the bond angle and is inversely related to the bond length. The bond lengths corresponding to the tetrahedral and octahedral sites have been intended using the following relation^75^:

16 $$ {\text{d}}_{{{\text{A}} - {\text{OA}}}} = {\text{ a }}\left( {{\text{u}} - \frac{1}{4}} \right)\sqrt 3 $$

17 $$ {\text{d}}_{{{\text{B}} - {\text{OB}}}} = {\text{ a}}\sqrt {2\left( {{\text{u}} - \frac{3}{8} } \right)^{2} { } + \left( {{ }\frac{5}{8}{ }{-}{\text{ u}}} \right)^{2} { }} $$

The bond length of tetrahedral site d _**A-OA**_ increases as the concentration of Ag^2+^ increases, this happened due to the expansion of the tetrahedral A-site as some of the cobalt ions from the B-site move towards the A-site. On the length the other hand, the octahedral bond d _B-OB_ decreases by the increasing concentration of Ag^2+^, the reason is a substitution of the Ag ion with the Co ion that has smaller ionic radii as compared to the silver ion therefore, shrinkage at the B-site.

The interatomic distance between cation–cations (*b–f*) and cation–anion (*p–s*) has been calculated using experimentally calculated lattice constant (***a***) and oxygen parameter (***u***) as given below and their values are tabulated in Table 5.

**Table 5:** Different structural parameters, i.e. bond angles, bond lengths, and hoping length, were determined to determine the effect of doping on the cationic distribution.

Ag ion conc	0	0.05	0.1	0.15
L_a_ (Å)	3.6338	3.6397	3.6340	3.6319
L_b_ (Å)	2.9670	2.9718	2.9671	2.9654
d_A–OA_	0.6606	0.6607	0.6615	0.6629
d_B–OB_	2.9182	2.9278	2.9217	2.9185
b	2.9670	2.9718	2.9671	2.9654
C	3.4791	3.4847	3.4793	3.4773
D	3.6338	3.6397	3.6340	3.6319
E	10.9016	10.9192	10.9020	10.8958
F	5.1390	5.1473	5.1392	5.1363
P	2.7627	2.7706	2.7652	2.7625
Q	0.6656	0.6607	0.6615	0.6629
R	1.2745	1.2651	1.2667	1.2695
S	3.2500	3.2533	3.2488	3.2476
θ_1_	91.0955	91.0692	91.0451	91.0226
θ_2_	105.7591	105.1553	104.608	104.1095
θ_3_	78.2615	78.6809	79.2288	79.7122
θ_4_	90.4574	90.4605	90.4636	90.4667
θ_5_	96.5787	95.9209	95.3485	94.8472

#### Cation–cation distances

The lengths between the A and B cations have been calculated by the following relation^45^:

18 $$ {\text{c}} = \frac{{a\sqrt {11} }}{8} $$

19 $$ {\text{e}} = \frac{a3\sqrt 3 }{8} $$

The bond length between the B–B cations can be estimated by^76^:

20 $$ {\text{b }} = \frac{a\sqrt 2 }{4} $$

21 $$ {\text{f }} = \frac{a\sqrt 6 }{4} $$

The bond lengths of A-A cations can be intended by using the following relation^47^:

22 $$\mathrm{d }=\frac{a\sqrt{3}}{4}$$

#### Cation–anion distances

The shorter edge bond lengths between A–O and B–O atoms are denoted by ‘q’ and ‘p’ respectively. The lengths of the bonds between A–O and B–O atoms at the larger edges are symbolized by ‘r’ and ‘s’, respectively. The shortest and largest bond lengths between cations (A, B) and anions O have been estimated by the following equations^77^:

23 $$ {\text{p}} = {\text{a}}\left( {\frac{5}{8} - {\text{u}}} \right) $$

24 $$ {\text{q}} = {\text{a}}\sqrt 3 \left( {{\text{u}} - \frac{1}{4}} \right) $$

25 $$ {\text{r}} = {\text{a}}\sqrt {11} \left( {u - \frac{1}{4}} \right) $$

26 $$ {\text{S}} = {\text{a}}\sqrt 3 \left( {\frac{u}{3} + \frac{1}{8}} \right) $$

For the computation of bond angles among the cations and between the cation and anion, the following relations are used^78^:

27 $$ \uptheta_{{1}} = {\text{ cos}}^{{ - {1}}} \left( {\frac{{p^{2} + q^{2} - c^{2} }}{2pq}} \right) $$

28 $$ \uptheta_{{2}} = {\text{ cos}}^{{ - {1}}} \left( {\frac{{p^{2} + r^{2} - e^{2} }}{2pr}} \right) $$

29 $$ \uptheta_{{3}} = {\text{ cos}}^{{ - {1}}} \left( {\frac{{2p^{2} - b^{2} }}{{2p^{2} }}} \right) $$

30 $$ \uptheta_{{4}} = {\text{ cos}}^{{ - {1}}} \left( {\frac{{p^{2} + s^{2} - f^{2} }}{2ps}} \right) $$

31 $$ \uptheta_{{5}} = {\text{ cos}}^{{ - {1}}} \left( {\frac{{r^{2} + q^{2} - d^{2} }}{2rq}} \right) $$

From calculated band angle values, we observed that on account of Ag substitution θ_3_ and θ_4_ angle increases, while θ_1_, θ_2,_ and θ_5_ decrease**.** This variation shows that the interaction between the B-B site reduces hence θ_3_ and θ_4_ angles increase while θ_1_, θ_2,_ and 5 decrease gradually, because of the increase in magnetic interactions among the A-B and A-A site.

### FTIR analysis

**Figure 9:**
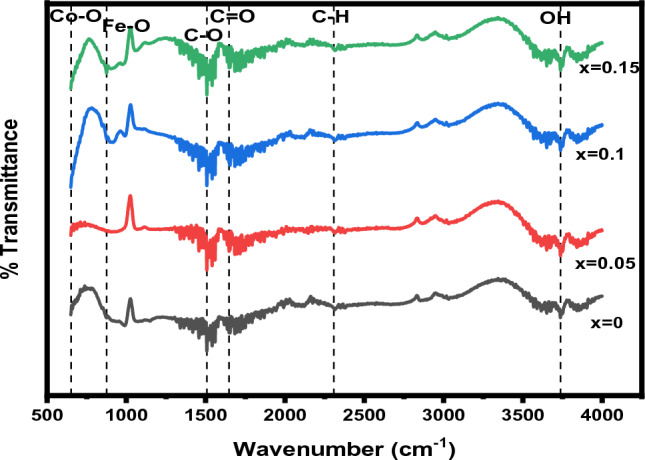
FTIR spectra of Ag_x_Co_1−x_Fe_2_O_4_ nanoparticles with various concentrations.

The presence of oxygen-based functional groups and their responses can be calculated through FTIR spectroscopy. The FTIR spectra of Ag_x_Co_1−x_Fe_2_O_4_ with various concentrations (x = 0, 0.05, 0.1, 0.15) synthesized by the hydrothermal technique in the range of 500–4000 cm^−1^ are depicted in Fig. 9. All bond peaks are expressed in Table 6. It has been analyzed from the figure that the band at 3736.16 cm^−1^ is attributed to the OH stretching vibration of the free alcoholic group^79^ and the band at 2305.83 cm^−1^ is assigned to stretching vibrations of the C–H bond^80^. Similarly, the bands associated with wavelengths 1649.53 cm^−1^ and 1504.61 cm^−1^ appear due to vibrational stretching of C=O and C–O bonds^81,82^. The absorption band in the range 1100–1300 cm^−1^ corresponds to the NO^3−^ ions vibration^83^. Furthermore, the peaks related to the wavelengths of 874.65 cm^−1^ and 651.5 cm^−1^ correspond to Fe–O and Co–O stretching vibrations of the tetrahedral and octahedral sites, respectively, confirming the formation of the inverse spinel structure of the characterized Ag_x_Co_1−x_Fe_2_O_4_ nanoparticles^79^.

**Table 6:** FTIR bands observed by various techniques synthesized from Ag_x_Co_1−x_Fe_2_O_4_ nanoparticles.

Observed bands	Bonds	Stretching/bending
3736.16 cm^−1^	–OH	Stretching vibration of free alcoholic group
2305.83 cm^−1^	C–H	Stretching vibration
1649.53 cm^−1^	C=O	Stretching vibration of a carbonyl group
1504.61 cm^−1^	C–O	Stretching vibration
1288.41 cm^−1^	NO^3−^	Stretching vibration
874.65 cm^−1^	Fe–O	Stretching vibration of tetrahedral site
651.5 cm^−1^	Co–O	Stretching vibration of octahedral site

### UV–Vis analysis

The optical properties of Ag_x_Co_1−x_Fe_2_O_4_ nanoparticles prepared by hydrothermal technique with different concentrations (x = 0, 0.05, 0.1, and 0.15) have been investigated by UV–visible spectroscopy. For UV–vis spectroscopy, each synthesized sample was dissolved in 4 ml of deionized water. The absorption spectra of all samples were recorded in the wavelength range of 200–1000 nm. The parameters that affect the absorbance value of any material are band gap, surface roughness, grain size, lattice parameters, and impurities^84^. The band gap energy of the samples can be estimated by using the Tauc relation^85^:

32 $$ \left( {\upalpha {\text{h}}\upnu } \right)^{{\text{n}}} = {\text{ A}}\left( {{\text{h}}\upnu - {\text{E}}_{{\text{g}}} } \right)^{{\text{n}}} $$

**Figure 10:**
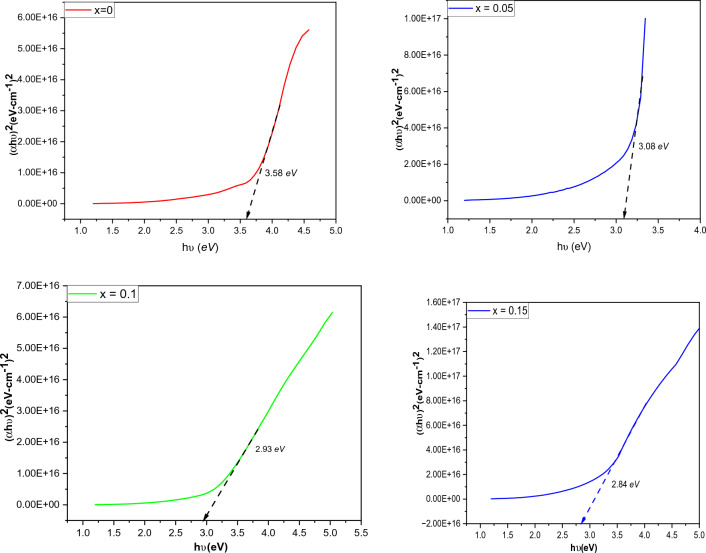
Determination of the optical band gap for Ag_x_Co_1−x_Fe_2_O_4_ nanoparticles with various concentrations (x = 0, 0.05, 0.1, and 0.15).

Here, ‘E_g_’ refers to the optical band gap energy, and ‘h’ belongs to Planck’s constant (6.62 × 10^–34^ J/s)^52,86^. The band gap energies of the nanoparticles with different concentrations of dopant have been measured by plotting graphs between direct band gap (αhν)^2^ and photon energy (hν) illustrated in Fig. 10.

The energy band gap values depicted for prepared nanoparticles with concentrations x = 0, 0.05, 0.1, and 0.15 are 3.58 eV, 3.08 eV, 2.93 eV, and 2.84 eV, respectively. The band gap values are in the range of 0–4 eV which reveals that the analyzed material has semiconductor properties^87^. It is depicted from the graph that the value of Eg increases as the concentration of substituent Ag^2+^ increases in the material. It is generally known that when the particle size decreases, the band gap energy increases usually^88^. This may be due to the fact that whenever the crystallite size reaches to nanoscale; where all the elements are made up of a finite number of atoms, the electron–hole pair becomes very close; thus, the columbic force is neither to be neglected longer, which may result in overall higher kinetic energy. However, a larger band gap indicates that the large energy is mandatory for the excitation of an electron from the valence to the conduction band. At a concentration of x = 0.05 concentration, both crystallite size and band gap energy have been observed to increase: probably due to some interfacial defects and the development of energy levels^89^.

#### BET analysis

**Figure 11:**
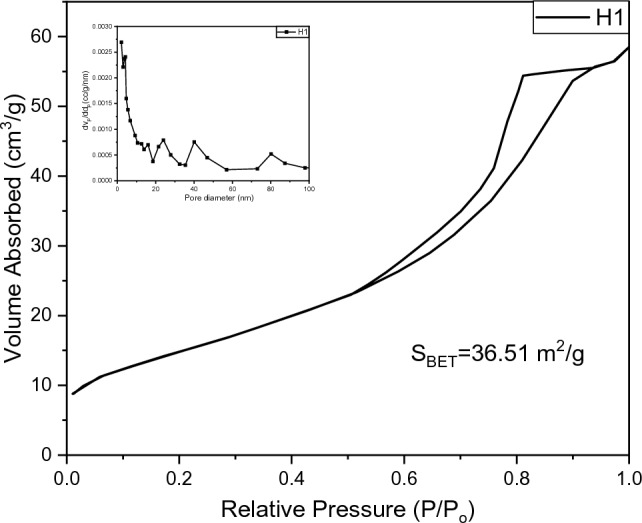
Pore size distribution (in set) with the BET isotherm of COF.

The BET analysis of the synthesized material is shown in Fig. 11. The surface textural characterization was analyzed through adsorption properties. From the figure, it can be seen that the nitrogen adsorption–desorption isotherm of CoFe_2_O_4_ against relative pressure P/P_o_ exhibited a hysteresis loop. According to the IUPAC classification, it can be assigned to type IV which contains large and micropores. According to the IUPAC classification, the graph displayed an H2-type hysteresis loop. From the pore size distribution graph, it has been observed that the pore width range is 2–95 nm.

Various methods were used to synthesize COF and analyzed through BET analysis. In Table 7 below, various values of S_BET_ are presented from the literature.

**Table 7:** Pastly synthesized COF with S_BET_ values.

Material name	S_Bet_ (m^2^/g)	Methodology
CoFe_2_O_4_^105^	173	Hydrothermal
CoFe_2_O_4_^47^	145	Chemical precipitation method
CoFe_2_O_4_^106^	140	chemical coprecipitation technique
CoFe_2_O_4_^107^	76	Solvothermal synthesis
CoFe_2_O_4_^108^	30	combined sonochemical and co-precipitation technique
CoFe_2_O_4_^109^	16	one-pot synthesis

**Figure 12:**
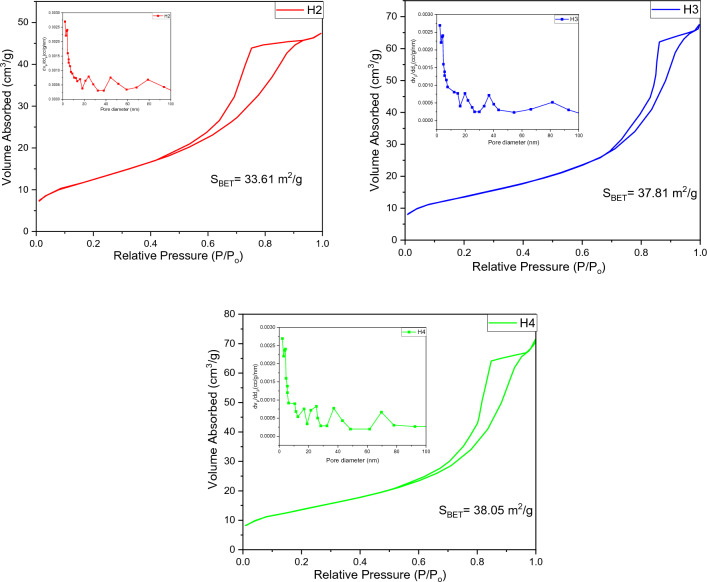
Pore size distribution (in set) with the BET isotherm of Ag-doped COF.

In Fig. 12 the Ag-doped COF is depicted with isotherm and pore size distribution. By utilizing the BJH (Barrett–Joyner–Halenda) method the pore size distribution is calculated and figured out in Fig. 12.

### VSM analysis

**Figure 13:**
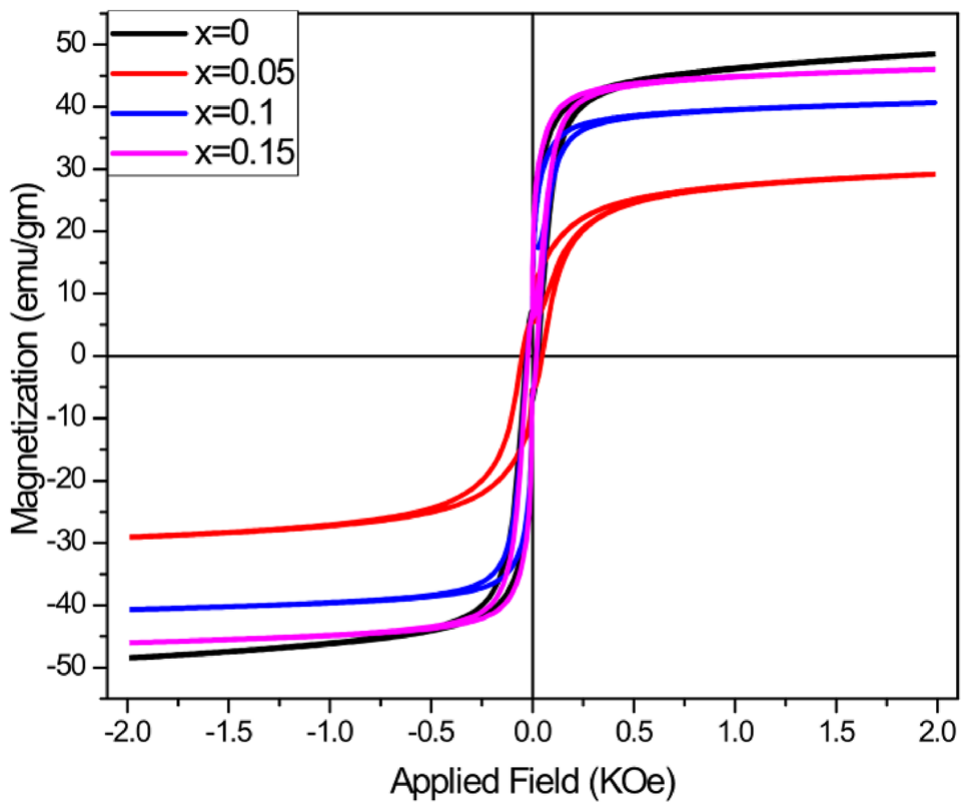
Hysteresis loop of Ag_x_Co_1−x_Fe_2_O_4_ nanoparticles with different concentrations x = 0, 0.05, 0.1, and 0.15.

The hysteresis loop of Ag_x_Co_1−x_Fe_2_O_4_ nanoparticles with different concentrations is shown in Fig. 13. The values of saturation magnetization, coercivity, remanence, magnetic anisotropy, and magnetic moment of all samples are listed in Table 6. It is long-familiar that cobalt ferrite nanoparticles show an inverse spinel structure in which ferric and cobalt ions occupy both the tetrahedral (A-site) and octahedral (B-site). The net magnetization of the ferromagnetic spinel structure was observed due to the difference in magnetic moments of the A and B site sub-lattices^90^.

The saturation magnetization decreases to x = 0.05 at the concentration but increases gradually at higher concentrations. This change in saturation magnetization is observed by replacing Co^2+^ ions with nonmagnetic Ag^2+^ ions. The magnetic moment of iron is 5 μB, and the octahedron and tetrahedron spots are distributed equally. Cobalt ions reach an octahedral point with a magnetic moment of 3 μB, and silver has a larger orbital orbit of 0 μB, which prefers to occupy B point and push some cobalt ions to point A. Consequently, saturation magnetization decreases because Co^2+^ ions in the octahedral point are replaced by Ag^2+^ ions, which reduces the magnetic moment in the B octahedral point. When the Ag content is high, silver ions can no longer dissolve in the network and form the second phase of silver ions on the grain boundaries. The formation of secondary phases can lead to an increase in Co^2+^ ions in the octahedron site, thus increasing saturation magnetization. Thus, the net magnetic moment of the ferric and cobalt ions is observed. The remaining magnetic resonance shows that the magnetism remains in the medium after the external magnetic field is removed. In Table 8, various magnetic parameters of cobalt ferrites are presented.

**Table 8:** Various parameters of Ag-doped cobalt ferrites.

Material name	Ms (emu/g)	Mr (emu/g)	Hc (Oe)	Mr/Ms	Methodology
Ag_x_Co_1−x_Fe_2_O_4_^101^	37.55	13.65	1350	0.36	Solgel method
Ag_x_Co_1−x_Fe_2_O_4_^43^	28.89	10.64	653.25	2.7	Green synthesis
(Ag_x_Ni_0.4_Co_0.6-x_Fe_2_O_4_)^110^	44.29	33.09	2029.38	0.75	Green and chemical route
Ag_x_Co_1−x_Fe_2_O_4_^111^	45.5	18	672	0.3956	Green and chemical route
Ag_x_Co_1−x_Fe_2_O_4_^32^	32.93	17.27	971.68	0.52	Auto combustion method
Ag_x_Co_1−x_Fe_2_O_4_^112^	35.52	5.49	151.24	0.104	Sol–gel method

**Figure 14:**
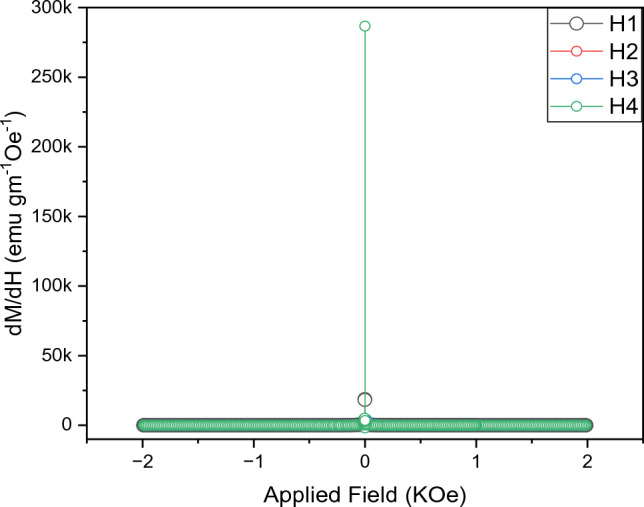
Change in magnetization in response to the applied magnetic field.

The way materials can respond to the applied magnetic field can be seen through changes in magnetization in Fig. 14. From the figure, it is concluded that the synthesized MNPs have a single magnetic phase. The peak height and shape of the dM/dH curve reveal that the magnetic nanoparticles are surrounded by a shell of a non-magnetic layer, whereas the peak width of the dM/dH curve reflects the particle size distribution. The narrow sharp width of the CoFe2O4 dM/dH curve (x = 0) narrates that the magnetic core is surrounded by a nonmagnetic shell, while the broad peak width of the dM/dH curve with concentrations x = 0.05, 0.1 and 0.15 demonstrates the large particle size dispersion. Although the magnetic hysteresis manifests a low coercive field, its derivative illustrates a broad peak, which strongly suggests the presence of a secondary phase in the materials used in the magnetic coupling.

The squareness ratio (**M**_**r**_**/M**_**s**_) has been calculated by using an M–H hysteresis loop. It seems that the value of the squareness ratio is less than 0.5, which conflicts with the uniaxial magneto crystalline anisotropy of synthesized nanoparticles^91^.

Using the law of saturation (LAS) and the magnetization portion (1 T H ≤ 2 T), when the magnetization reached the saturated value, the uniaxial magneto crystalline anisotropic constant K_u_ has been calculated for the prepared Ag_x_Co_1−x_Fe_2_O_4_ nanoparticles. The Modified Morish Law of Saturation is as follows^92^:

33 $$ {\text{M}} = {\text{ M}}_{{\text{S}}} \left( {1 - \frac{B}{{H^{2} }}} \right) $$

where B is a parameter obtained through a fitting process. Equation (34) is used to evaluate the values of K_u_, and the results are listed in Table 8. It has been found that the particle size of the prepared nanoparticles affects magnetic anisotropy. The spin–orbit coupling, crystal field interactions, and their ratio are the causes of uniaxial magnetic anisotropy. Uniaxial magneto-crystalline anisotropy originates due to spin–orbit coupling caused by unquenched metal ions at the B sites of MFe_2_O_4_ ferrites^93,94^.

34 $$ {\text{K}}_{{\text{u}}} = {\text{ M}}_{{\text{s}}} \sqrt{\frac{15B}{4}}  $$

The magnetic anisotropy field (H_a_) of the prepared samples is estimated with the help of the following relation^95^

35 $$ {\text{H}}_{{\text{a}}} = \frac{{2K_{u} }}{{{\text{Ms}}}} $$

The magnetocrystalline anisotropy constant was computed in order to understand the function of magnetic anisotropy. Coercivity affects the magnetic anisotropy constant's value. As a result, its larger value is associated with the strong resistance of dipoles to annihilation when a reverse magnetic field is applied. Meanwhile, it is believed that the lower value of the anisotropy constant results because of the relaxation of the magnetization for particles with lower anisotropy due to the influence of interparticle interactions.

Two kinds of the magnetic moment have been calculated i.e., the calculated magnetic moment and the observed magnetic moment. The observed magnetic moment has been estimated from the hysteresis loop, with the help of the following relation^60,96,97^:

36 $$ {\text{n}}_{{\text{B}}} = \frac{{{\text{M }} \times {\text{Ms}}}}{5588} $$

where M is molecular weight and ‘M_S_’ refers to saturation magnetization. On account of Neel’s two sublattice model, calculated magnetic moment was computed by using the relation^98^:

37 $$ {\text{M}}_{{({\text{Calc}})}} = {\text{ M}}_{{\text{B}}} \left( {\text{x}} \right) \, {-}{\text{ M}}_{{\text{A}}} \left( {\text{x}} \right) $$

where ‘M_A_’ corresponds to the magnetic moment of the A-sublattice and ‘M_B_’ represents the B-sublattice, respectively.

The calculated magnetic moment of silver-substituted cobalt ferrite is calculated according to the estimated cationic distribution as follows:

At **x = 0**:

$$ \begin{gathered} {\text{M}}_{{({\text{Calc}})}} = \, \left[ {{\text{Co }}\left( {1} \right){\text{ Fe }}\left( {1} \right)} \right]^{{{\text{Octa}}}} {-} \, \left[ {{\text{Fe }}\left( {1} \right)} \right]^{{{\text{tetra}}}} \hfill \\ {\text{M}}_{{({\text{Calc}})}} = \, \left[ {\left( {{1} \times {3}} \right) \, + \, \left( {{1} \times {5}} \right)} \right]^{{{\text{Octa}}}} {-} \, \left[ {\left( {{1} \times {5}} \right)} \right]^{{{\text{tetra}}}} \hfill \\ {\text{M}}_{{({\text{Calc}})}} = { 8 }{-}{ 5} \hfill \\ {\text{M}}_{{({\text{Calc}})}} = { 3}\upmu {\text{B}} \hfill \\ \end{gathered} $$

Similarly, a magnetic moment at **x = 0.1:**

$$ \begin{gathered} {\text{M}}_{{({\text{Calc}})}} = \, \left[ {{\text{Ag }}\left( {0.0{5}} \right){\text{ Co }}\left( {0.{93}} \right){\text{ Fe }}\left( {{1}.0{2}} \right)} \right]^{{{\text{Octa}}}} {-} \, \left[ {{\text{Co }}\left( {0.0{2}} \right){\text{ Fe }}\left( {0.{98}} \right)} \right]^{{{\text{tetra}}}} \hfill \\ {\text{M}}_{{({\text{Calc}})}} = \, \left[ {\left( {0.0{5} \times 0} \right) \, + \, \left( {0.{93} \times {3}} \right) \, + \, \left( {{1}.0{2} \times {5}} \right)} \right]^{{{\text{Octa}}}} {-} \, \left[ {\left( {0.0{2} \times {3}} \right) \, + \, \left( {0.{98} \times {5}} \right)} \right]^{{{\text{tetra}}}} \hfill \\ {\text{M}}_{{\left( {{\text{Calc}}} \right) }} = { 4}.{96 }{-}{ 7}.{89} \hfill \\ {\text{M}}_{{({\text{Calc}})}} = { 2}.{93}\,\,\upmu {\text{B}} \hfill \\ \end{gathered} $$

**Figure 15:**
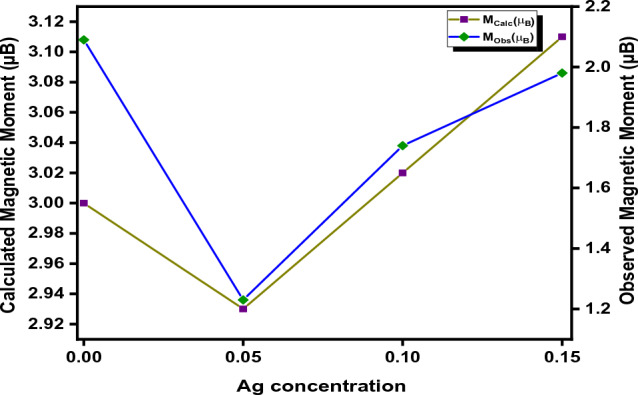
Graph between the calculated and observed magnetic moment.

It is evident that the values of calculated magnetic moment at tetrahedral A-site increase and due to the substitution of non-magnetic Ag^2+^ ion at octahedral B-site decrement in magnetic moment has been observed. Figure 15 revealed that the calculated magnetic moment ‘M_(Calc)_’ has good agreement with the observed magnetic moment ‘n_B_’ both decrease slightly; but for higher concentrations of the dopant, the magnetic moment again increases because the non-magnetic Ag^2+^ ion was not dissolved further in lattice results in the increment in magnetic moment caused by cobalt and ferric ions. The Co^2+^ ions at the octahedral B-site are replaced by the non-magnetic Ag^2+^ ions, and some of the cobalt ions move toward the tetrahedral A-site. The difference in sublattice magnetizations caused by the antiferromagnetic interaction between the spins on both sides constitutes the net magnetic moment per formula unit at zero Kelvin. Neel's two-sublattice ferrimagnetism model predicts that the magnetic moments of ions on the tetrahedral (A) and octahedral (B) sites are aligned antiparallel to one another, indicating that their spins have a collinear structure. According to Neel’s sublattice model, it is observed that magnetization at the octahedral B-site reduces; the consequence is the weakening of the B–B interaction. Therefore, the overall magnetization in the system was estimated as a result of the presence of iron and cobalt ions. According to the Yafet Kittle model, the replacement of Ag^2+^ ion (non-magnetic) by Co^2+^ ion at the octahedral B site reduces the magnetic moment of the respective site, hence the reduction of A-B interaction is observed. However, the ionic radius of silver is greater than that of cobalt; therefore, it forcefully shifts some cobalt ions towards the tetrahedral A-site due to this A-A interaction increases for higher concentration which results in again increase in an increase magnetic moment.

Regarding the findings in Table 9, it is evident that the values of the coercivity, remanence, and squareness ratios exhibit the saturation magnetization trend, i.e., For x = 0.05, saturation magnetization suddenly decreases due to the substitution of nonmagnetic ions; after that, with increasing concentrations of Ag^2+^ ions in cobalt ferrite nanoparticles, the saturation magnetization values gradually increase.

**Table 9:** Magnetic parameters extracted from the hysteresis loop.

Dopant conc. (x)	0	0.05	0.1	0.15
Saturation magnetization M_S_ (emu/g)	48.34	29.06	40.69	45.85
Coercivity H_C_ (Oe)	0.03	0.0503	0.0223	0.0215
M_r_/M_s_	0.2066	0.2240	0.1999	0.2178
M_s_ (emu/g) (LAS)	49.54	30.55	41.08	46.46
Retentivity M_r_ (emu/g)	9.99	6.51	8.135	9.99
K_u_ × ${10}^{2}$ (erg/cm^3^)	9.8610	7.2792	6.4501	4.8518
Magnetic moment Obs. (µ_B_)	2.09	1.23	1.74	1.98
Magnetic moment Cal. (µ_B_)	3	2.93	3.02	3.11

Additionally, a substantial hysteresis loop shift has been observed and associated with exchange bias events. One way to describe the exchange bias field is as follows:

38 $$\mathrm{H }_{\text{EB}}=\frac{-[H\left(-\right)+H\left(+\right)]}{2}$$

where H ( +) and H (−) are the magnetization's intercepts with + ve and −ve on the field axis, respectively. H_EB_ has a maximum value of 1.98 Oe for AgCoFe_2_O_4_ and a minimum value of 1.8 Oe for CoFe2O4. The H2, H3, and H4 have the values (1.55, 1.75, 1.85) for higher intercept and (1.40, 1.63, 1.71) for lower intercept. As the magnetic structure of the surface differs from the one found in the core, this tendency may be explained by the presence of several spin configurations in the nanostructure. However, this behavior is attributed to the interaction between the weak antiferromagnetic spins at the core of the Ag-ferrite grains and the weak ferrimagnetic spins on their surface. This is compatible with the core–shell hypothesis of the grain structure.

The switching field distribution (SFD) is depicted as:

39 $$\mathrm{SFD }=\frac{\mathrm{\Delta H}}{\mathrm{Hc}}$$

SFD denotes the rectangularity of the H–M loop and ΔH is estimated from the half-width of the peak of the dM/dH curve. Because of the intrinsic magnetic properties of crystallinity and substitution uniformity, SFD exists.

There has been observed to be a direct impact of magnetization on antibacterial activity^99^. Because of the presence of oxides in the composite, a magnetic moment is observed in the magnetic properties, and because of the magnetic properties, it attracts the membrane of bacteria. It has been noticed that particle size is the main reason for antibacterial activity and from the antibacterial results it can be seen that the nanoparticles have high antibacterial results.

### Bacterial microflora

In LBA (Luria Bertani Agar) media, diseased Ag-substituted cobalt ferrite samples were inoculated. After incubation for 24 h at 25 °C, the bacterial growth was clearly visible. By using the streaking method, the bacterial colonies were purified in separate plates.

**Figure 16:**
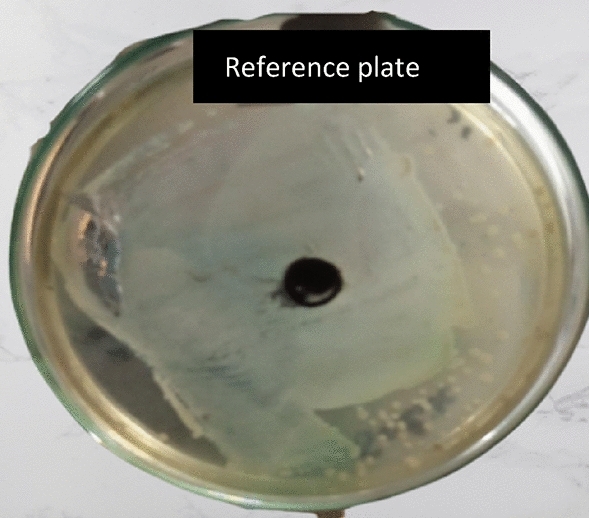
The reference plate and bacterial growth is detected on the reference plate.

**Figure 17:**
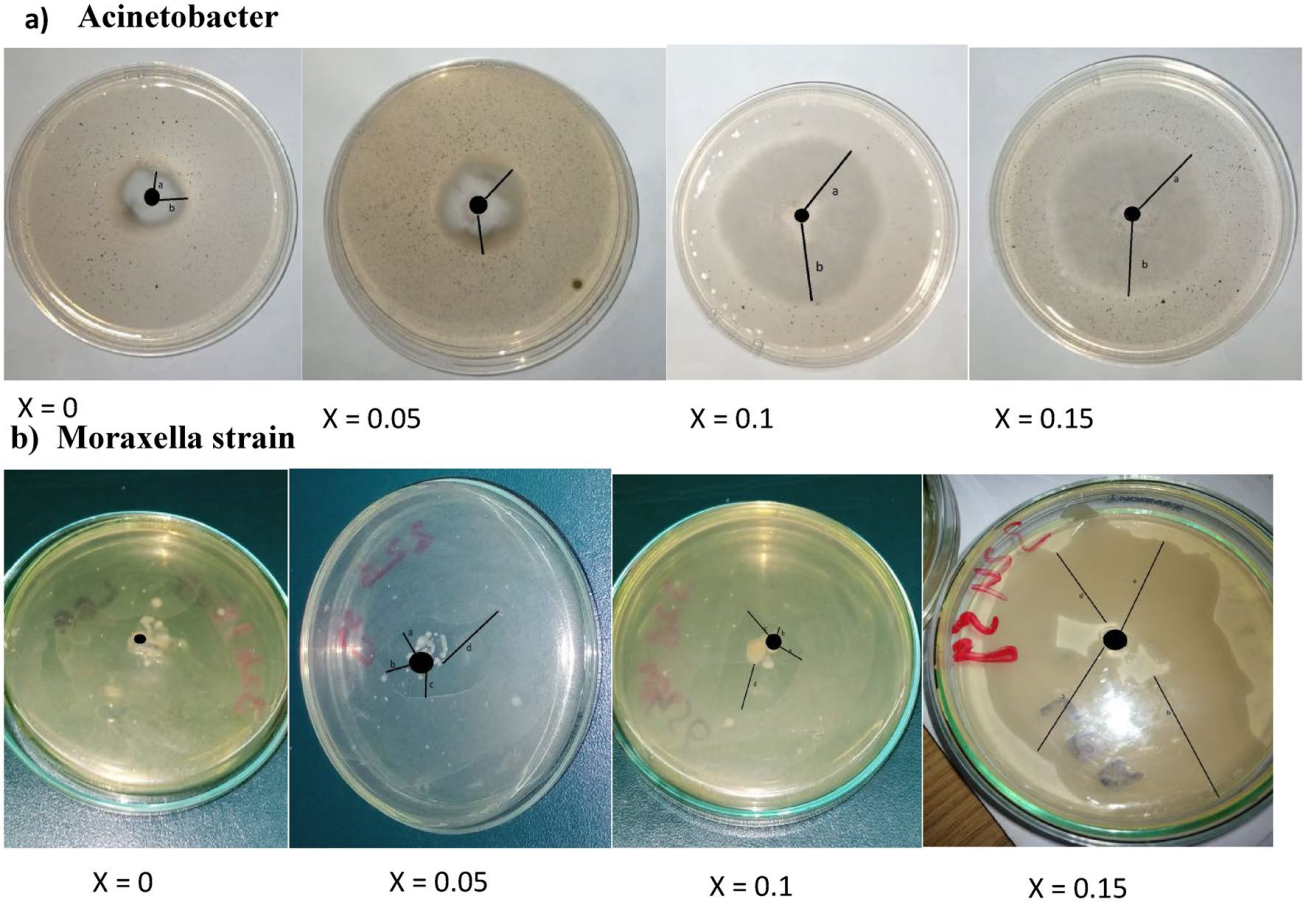
The results of H_1_, H_2_, H_3_, and H_4_ in Acinetobacter Lwoffii (**a**) and Moraxella species (**b**).

Ag^2+^ doped cobalt ferrite nanoparticles have been prepared using the hydrothermal method and their antibacterial activity against Acinetobacter Lwoffii which is a cause of chronic diseases such as diabetes mellitus, renal disease, heavy smoking, etc.) and Moraxella species, which is a cause of diseases such as blood and eye infection). Different concentrations of Ag-doped cobalt ferrites [H_1_(x = 0), H_2_(x = 0.05), H_3_(x = 0.1), and H_4_(x = 0.15)] nanoparticle powder sample were used against Acinetobacter Lwoffii and Moraxella species to check inhibition zones. For this purpose, the good diffusion method was used. Figure 16 is used as a reference and now activity is observed on the plate, while antibacterial activity can be depicted in Fig. 17. From Fig. 16 it can be seen that the bacteria spread all over the plate. But in Fig. 17, it can be observed that the nanoparticles start to kill bacteria, and a clear image of bacteria-killing is observed in Fig. 17.

**Figure 18:**
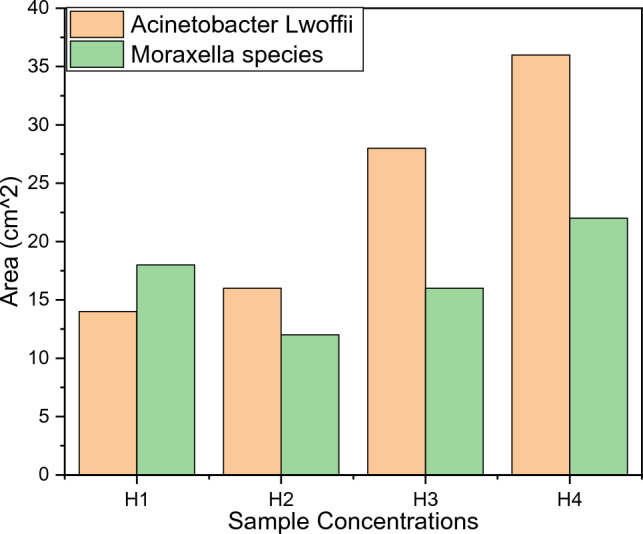
Inhibition zones of H1, H2, H3, and H4 against Acinetobacter Lwoffii and Moraxella species.

The overall dimensions of the glass Petri dish were 10 × 10 × 2 cm. The inhibition zone of Moraxella strain with Ag-doped cobalt ferrites nanoparticles of H_1_, H_3_, and H_4_ sample was average length of 2.7 cm or 15 mm, 3.7 cm or 37 mm, 4.72 cm or 47.2 mm, and 4.9 cm or 49 mm approximately and the inhibition zone of Acinetobacter Lwoffii with Ag-doped cobalt ferrites nanoparticles of H_1_, H_3_, and H_4_ sample was average length 2.5 cm or 25 mm, 4.5 cm or 45 mm, 5.3 cm or 53 mm, and 6.9 cm or 69 mm approximately (Fig. 18).

The study corroborated the existence of pathogenic microorganisms. The isolated and identified were Pseudomonas syringae and Bacillus species*.*

For the eudaemonia of individuals and societies, some of the medicative plants have been of keen interest. From the figures it has been observed that the samples H_4_ and H_3_ show antibacterial activity against Pseudomonas syringae and the inhibition zone values are calculated, which is very high concentrated values against Pseudomonas syringae. On the other hand, the concentration sample H_1_ and H_2_ has no effect on Pseudomonas syringae, which is suggested due to the low concentration of Ag in cobalt ferrite. On the other hand, the high concentration of Ag in cobalt ferrite may not have an effect on Bacillus species, and a lower concentration shows antibacterial activity results on Bacillus species.

## Conclusion

Silver-doped cobalt ferrite (Ag_x_Co_1−x_Fe_2_O_4_) nanoparticles with different concentrations of Ag^2+^ (x = 0, 0.05, 0.1, 0.15) are prepared through hydrothermal technique. XRD analysis substantiates the formation of the cubic inverse spinel structure. FTIR analysis confirmed the formation of an inverse spinel structure with the major band at 874 cm^−1^, which could be due to the stretching vibrations of a metal–oxygen bond. The optical band gap energy increases as the concentration of Ag increases as a result of a decrease in the crystallite size. The saturation magnetization decreases initially, but as the concentration of silver increases, saturation magnetization increases. Antibacterial activity testing against Acinetobacter Lwoffii and Moraxella was observed. It was concluded that the nanoparticles with a high concentration of silver are more effective for bactericidal activity against the Acinetobacter Lwoffii bacterial strain, while for killing the Moraxella bacterial strain nanoparticles of higher concentration are effective and kill the bacteria. This material is useful for memory storage and antibacterial activity.

## Data Availability

The data sets used and/or analyzed during the current study are available from the corresponding author upon reasonable request.
